# Y-Box Binding Protein 1 Regulates Angiogenesis in Bladder Cancer via miR-29b-3p-VEGFA Pathway

**DOI:** 10.1155/2021/9913015

**Published:** 2021-07-01

**Authors:** Dongyang Gao, Qian Niu, Yuwen Gong, Qi Guo, Su Zhang, Yuhan Wang, Shanhui Liu, Hanzhang Wang, Robert Svatek, Ronald Rodriguez, Junhai Ma, Zhiping Wang

**Affiliations:** ^1^Institute of Urology, Lanzhou University Second Hospital, Key Laboratory of Urological Diseases in Gansu Province, Gansu Nephro-Urological Clinical Center, Lanzhou 730030, Gansu, China; ^2^Department of Pathology, Lanzhou University Second Hospital, Lanzhou, China; ^3^Department of Urology, University of Texas Health Science Center San Antonio, San Antonio, TX, USA

## Abstract

Angiogenesis plays a vital role in the development of bladder cancer (BC). The Y-box-binding protein 1 (YB-1) is a well-known oncoprotein which is closely related to angiogenesis of tumors, but the relationship and mechanism of YB-1 and angiogenesis in BC remain unclear. Based on 56 clinical BC specimens, this study found that high expression of YB-1 samples demonstrated a higher expression of vascular endothelial growth factor A (VEGFA) than those of YB-1 low expression. Subsequently, the expression of YB-1 and miR-29b-3p was regulated in the BC cell lines where we noted that YB-1 promoted VEGFA expression by downregulating the expression of miR- 29b-3p. The ability of BC cells to induce angiogenesis decreased after YB-1 was knocked down. Moreover, the in vivo study further confirmed that YB-1 promotes angiogenesis in BC. Our findings enhance the understanding of how YB-1 promotes angiogenesis in BC and provide evidence for YB-1 as a therapeutic target of BC. Moreover, this may provide new inspiration for miRNAs replacement therapies.

## 1. Introduction

Bladder cancer (BC) is a prevalent malignant tumor threatening human health [1]. At present, surgery is the primary treatment approach for BC, based on pathological stages, supplemented by chemotherapy or immunotherapy. Nevertheless, this mode of therapy has the disadvantages of high cost and high recurrence [2]. Targeted therapy might provide a novel solution for the treatment of tumors, where oncogenes are important targets.

Y-box-binding protein 1 (YB-1) is a well-known oncoprotein regulating tumor cell growth, invasion, metastasis, drug resistance, and angiogenesis [3, 4]. Patients with higher expression of YB-1 in BC tissue showed lower overall survival rates [5]. Moreover, our previous work revealed that elevated expression of YB-1 was significantly correlated with high clinical *T* stage and pathological *T* stage in BC [6]. Therefore, YB-1 is a potential molecular target for developing novel therapeutic strategies for BC.

YB-1, a member of the cold-shock protein superfamily, binds both DNA and RNA exerting numerous functions, including regulating transcription and translation, pre-mRNA splicing, mRNA packaging, noncoding RNA processing, and DNA repair [7]. Shuai-Lai et al. discovered that YB-1 can downregulate the expression of miR-29b-3p by inhibiting the biogenesis of miR-29b-2 in glioblastoma multiforme [8]. Additionally, miR-29b is known as tumor-suppressing microRNAs (miRNAs), playing important roles in cancer at stages ranging from initiation to metastasis [9]. The modulation of YB-1 on mir-29b-3p might be a vital aspect of the YB-1 tumor-promoting effect. Here, we analyzed the target genes of miR-29b-3p in miRDB (http://www.mirdb.org) and identified 1034 predicted targets of hsa-miR-29b-3p in miRDB [10]. We further analyzed the enrichment of the first 200 target genes in KEGG-PATHWAY using the Functional Annotation Chart of DAVID Bioinformatics Resources 6.8 (https://david.ncifcrf.gov) [11]. Consequently, we found there were 10 genes in pathways of cancer. The most important one was vascular endothelial growth factor A (VEGFA). As we know, high expression of VEGFA facilitates tumor growth and metastasis by promoting angiogenesis [12]. Moreover, VEGFA is a vital marker of BC [13]. As such, we hypothesized that “YB-1 upregulates VEGFA expression by inhibiting the biogenesis of miR-29b-3p to promote angiogenesis in BC”.

This study regulated the expression of YB-1 and miR-29b-3p in the BC cell lines and demonstrated that YB-1 promotes VEGFA expression by downregulating the expression of miR-29b-3p in BC. These findings provide insights into the oncogenic role of YB-1 in angiogenesis and provide evidence for YB-1 as a therapeutic target of BC. Arguably, this study, for the first time, reported the effect of YB-1 on VEGFA from the perspective of regulating miRNAs. This might provide a new perspective on miRNAs replacement therapy.

## 2. Materials and Methods

### 2.1. Tissues and Clinical Data

The pathological information of radical cystectomy specimens between January 2017 and January 2018 was investigated to select BC specimens in the Pathology Department of Lanzhou University Second Hospital. Notably, bladder adenocarcinoma and squamous cell carcinoma were excluded. To analyze the expression of YB-1 and VEGFA, immunohistochemistry (IHC) was performed on all selected BC specimens. IHC studies on human BC samples were approved by the Lanzhou University Second Hospital Ethics Board.

### 2.2. Cell Culture

The human BC cell lines EJ, UMUC3, SW780, RT4, and the human endothelium cell line EA.hy926 were used. All cell lines were purchased from Cell Bank, Chinese Academy of Sciences. All cells were cultured in RPMI‐1640 medium (Gibco, Grand Island, NY) containing 10% fetal bovine serum (PAN, Germany) in an incubator with 5% CO_2_ at about 37°C.

### 2.3. Transfection and Construction of Stable Transformed Cell Lines

To knock down the expression of YB-1 in EJ cells with high expression of YB-1, pGPU6/GFP/Neo-YB-1-shRNA (target sequence, 5′- GAAGTACCTTCGCAGTGTAGG-3′) and pGPU6/GFP/Neo-NC-shRNA (target sequence, 5′-GTTCTCCGAACGTGTCACGT-3′; NC, negative control) were purchased from Shanghai GenePharma. EJ cells in a 24-well plate were transfected with mixtures containing different proportions of Lipofectamine 2000 (Shanghai GenePharma, China) and shRNA according to the manufacturer's protocol. Based on the proportion of green fluorescent cells, the transfection efficiency was measured under a fluorescence microscope 24–48 hr after the transfection. In all the cases, the transfection efficiency was less than 10%. Therefore, we constructed stable transformed cell lines using G418 (400 *μ*g/ml) and local trypsin digestion. After repeated selections, stable transformed cell lines with G418 resistance and GFP were eventually established. To knock down expression of YB-1 in UMUC3 cell line and miR-29b-3p in YB-1 knockdown EJ cell line, siRNA and miRNA inhibitors with 5-carboxyfluorescein (5-FAM) were purchased from Shanghai GenePharma. The sequences were listed as follows: YB-1-siRNA forward, 5′-GAAGUACCUUCGCAGUGUAGG-3′ and reverse, 5′- CCUACACUGCGAAGGUACUUC-3′; NC-siRNA forward, 5′-UUCUCCGAACGUGUCACGUTT-3′ and reverse, 5′- ACGUGACACGUUCGGAGAATT-3′; miR-29b-3p-Inhibitor, 5′- AACACUGAUUUCAAAUGGUGCUA-3′; NC-Inhibitor, 5′- CAGUACUUUUGUGUAGUACAA-3′. Cells of 65–75% confluence in cell culture dishes with a diameter of 6 cm were transfected by 200 pmol siRNA/inhibitors and 10 *μ*l Lipofectamine 2000 according to the manufacturer's protocol. Furthermore, we set up the MOCK group which was transfected only by 10 *μ*l Lipofectamine 2000. The knockdown efficiency was evaluated by RT-qPCR 48 hr after transfection and by Western Blot 72 hr after transfection.

### 2.4. RNA Extraction and RT-qPCR

Trizol reagent (Takara, Dalian, China) was used to extract the total RNA from all cells. mRNA and miRNAs were converted into cDNA by PrimeScript RT reagent Kit (Takara) and Mir‐X miRNA First-Strand Synthesis Kit (Takara), respectively. The RT-qPCR (Bio‐Rad CFX96) was performed by TB Green Premix Ex Taq (Takara). For data normalization, GAPDH and U6 (RNU6B) snRNA were used as endogenous control for mRNA and miRNAs, respectively. Relative gene expression was evaluated by the ΔΔCq method [14]. Sequences of the primers are listed in the Supplementary Materials (Table 1).

### 2.5. Western Blot

Cells in a 25 cm^2^ cell culture flask or cell culture dish with a diameter of 6 cm were trypsinized and washed with PBS. Subsequently, the centrifugation precipitate of the cells was lysed by 70–200 *μ*l of ice-cold RIPA Cell Lysis Buffer according to the precipitate volume. Cell lysates (10–15 *μ*l) were separated on 12% SDS–PAGE gel and electroblotted onto a nitrocellulose membrane. Antibodies against YB-1 (ab76149, Abcam, USA), VEGFA (19003-1-AP, Proteintech Group Inc., USA), GAPDH (60004-1-lg, Proteintech Group Inc., USA), and fluorescent secondary antibody against rat or rabbit (Licor, USA) were diluted 500, 500, 15000, and 15000 times to incubate nitrocellulose membrane, respectively. GAPDH served as endogenous control. Eventually, the analysis was performed by the Odyssey CLX near-infrared dual-color fluorescence imaging system.

### 2.6. Cell Proliferation Assay

Cell proliferation was assessed by Cell Counting Kit‐8 (CCK8; Dojindo Laboratories, Kumamoto, Japan) assay according to the manufacturer's protocol. About 5 × 103 cells were seeded in a 96‐well plate per well and cultured in a CO_2_ incubator. At each time point of 2, 24, and 48 hr after cells were seeded, the cell proliferation reagent was added to the 96-well plate and the cells were incubated 2 hr before the test. The time point of 2 hr is the basis point.

### 2.7. Colony Formation Assay

Cells were trypsinized, counted, and seeded in a 6-well plate with a density of 200 cells per well. The culture medium was replaced every 4 days. The culture was not terminated until the 14th day after seeding, when most cells formed visible colonies. Cells were fixed with 4% paraformaldehyde fixative and stained with GIMSA stain. Then, the colonies were counted and the colony formation rate was calculated with the equation: colony formation rate = (number of colonies/200) × 100%.

### 2.8. Tube Network Formation Assay

About 5 × 10^5^ cells of YB-1 knockdown EJ cell line (YB-1 KD-EJ) or control EJ cell line (CTRL-EJ) were seeded and cultured in cell culture dishes with a diameter of 6 cm, respectively. After attaching for 3 hr, the cells were washed twice with PBS and then cultured in serum-free RPMI‐1640 medium for 24 hr. Thereafter, the RPMI‐1640 medium in cell culture dishes was collected separately for instant use or storage at −20°C.

The cap strips for 8-strip PCR tubes were cut as the special Matrigel containers. After sterilization, these special containers were stuck to the bottom of the 96-well plate. Then, 10 *μ*l Matrigel was added to each container. The Matrigel was allowed to polymerize at 37°C for 1 hr. After that, the EA.hy926 cells were trypsinized, washed, and counted. Finally, the cells were resuspended with the serum-free cell culture medium collected previously and seeded in wells with the special Matrigel containers at a density of 1 × 10^4^ cells/100 *μ*l per well. Tube formation was optimal after 12 hr culture, and the images were captured. The numbers of grids and nodes formed by EA.hy926 cells in each well were calculated.

### 2.9. Tumor Formation in Nude Mice

Four-week-old male BALB/*c* nude mice of specific pathogen-free (SPF) grade were purchased from Beijing Vital River Laboratory Animal Technology Co., Ltd. (Beijing, China). All animal procedures conformed to the NIH Guide for the Care and Use of Laboratory Animals. The animal experiments were approved by the Animal Care Welfare Committee of Gansu University of Chinese Medicine.

The mice, divided into two groups (6 mice/group), were subcutaneously injected with YB-1 KD-EJ cells or CTRL-EJ cells (2  ×  10^6^ cells/0.15 ml). We measured the tumor length and width with external caliper every 3 days to calculate the tumor volume. Tumor volume was calculated by the formula: *V*  =  (length × width^2^)/2. Mice were sacrificed, and tumors were dissected when the tumors volume reached 2.0 cm^3^, or 40 days after inoculation.

### 2.10. Immunohistochemistry

IHC was performed via the streptavidin-peroxidase (SP) method. The procedure was performed strictly according to the reagent instructions, with a known positive section as a positive control and PBS as a negative control instead of an antibody.

The antigens were repaired by autoclave water bath. Antibodies against YB-1 (ab76149, Abcam, USA), VEGFA (19003-1-AP, Proteintech Group Inc., USA), and CD31 (#77699, CST, USA) for marking microvessels were all diluted 300 times to incubate pathological sections.

### 2.11. Immunohistochemical Analysis

To evaluate the expression of YB-1 and VEGFA, the proportion and intensity of positively stained cancer cells were evaluated. The estimated proportion of positively stained cancer cells corresponded to the positive rate score (1 = 0–25%, 2 = 26%–50%, 3 = 51%–75%, and 4 = 76%–100%). The intensity score represented an average estimated intensity of staining in positive cancer cells (0 = none, 1 = weak, 2 = moderate, and 3 = strong). Then, positive rate score timed intensity score to obtain the total score. We added the scores of the three different fields in one sample to obtain the IHC score. Based on the IHC score, the samples were evenly divided into the YB-1 high expression group and YB-1 low expression group. In instances of several samples with the same IHC scores at the middle, the pathologists would rejudge the expression levels of YB-1 in order to group them. Eventually, the difference of VEGFA expression between the two groups was analyzed.

### 2.12. Statistical Analysis

We used GraphPad Prism 8 Software to analyze statistical significance. For IHC scores, the significance was examined by Mann–Whitney U test. For others, the significance was examined by Student's *t*-test. *P* < 0.05 was considered statistically significant. The IHC scores were described by median ± quartile. Other data were expressed as mean ± SD (triplicate determination).

## 3. Results

### 3.1. YB-1 High Expression Samples Had Higher VEGFA Expression than Those of YB-1 Low Expression

**Figure 1:**
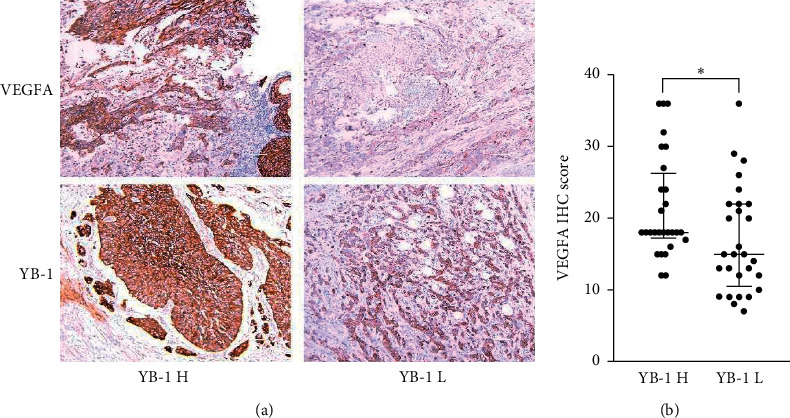
YB-1 and VEGFA immunohistochemical staining in BC tissues. YB-1 H, YB-1 high expression group; YB-1 L, YB-1 low expression group; IHC, immunohistochemistry;  ^*∗*^*P* < 0.05.

In total, 58 specimens of radical cystectomy were investigated. Two specimens were excluded due to pathological diagnosis of bladder adenocarcinoma and squamous cell carcinoma. Thus, 56 surgical specimens were included for IHC. Results showed that the YB-1 high expression group (YB-1 H) had higher VEGFA expression than that with YB-1 low expression (YB-1 L). The median of VEGFA IHC scores in YB-1 H and YB-1 L was 18 and 15, respectively (Figure 1).

### 3.2. The Expression of VEGFA Decreased While That of miR-29b-3p Increased, after YB-1 Was Knocked Down in BC Cell Lines

**Figure 2:**
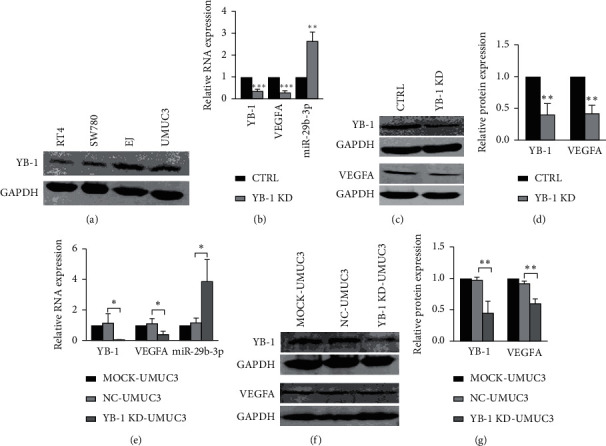
The expression level of YB-1 in four BC cell lines and the changes in expression of VEGFA and miR-29b-3p after knocking down YB-1 in EJ and UMUC3 cell lines. (a) The protein expression of EJ, UMUC3, SW780, and RT4 was measured by Western Blot. (b–d) The relative expression level of YB-1, VEGFA, and miR-29b-3p in CTRL-EJ and YB-1 KD-EJ cell lines was measured by RT-qPCR and Western Blot. (e–g) YB-1 was knocked down by siRNA in UMUC3 cells. Then the relative expression level of YB-1, VEGFA, and miR-29b-3p was measured by RT-qPCR and Western Blot. CTRL, control; YB-1 KD, YB-1 knockdown; NC, negative control; miR, microRNA;  ^*∗*^*P* < 0.05,  ^*∗∗*^*P* < 0.01, and  ^*∗∗∗*^*P* < 0.001.

First, the expression of YB-1 was determined in EJ, UMUC3, SW780, and RT4 BC cell lines. Consequently, the expression of YB-1 in the four cell lines was EJ > UMUC3 > SW780 > RT4 (Figure 2(a)). Next, the stable transformed cell lines of CTRL-EJ and YB-1 KD-EJ were effectively established. Results revealed that VEGFA expression was lowered by more than half, while miR-29b-3p expression doubled after YB-1 was knocked down in the EJ cell line (Figures 2(b)–2(d)). To confirm the generality of this situation in BC cell lines, YB-1 was further knocked down using siRNA in UMUC3. It is a similar case (Figures 2(e)–2(g)). Results indicated that YB-1 positively regulated VEGFA but negatively regulated miR-29b-3p.

### 3.3. After YB-1 Was Knocked Down, the Ability of EJ Cells to Induce Angiogenesis Weakened, and Colony Formation and Proliferation Ability Remained Unchanged

**Figure 3:**
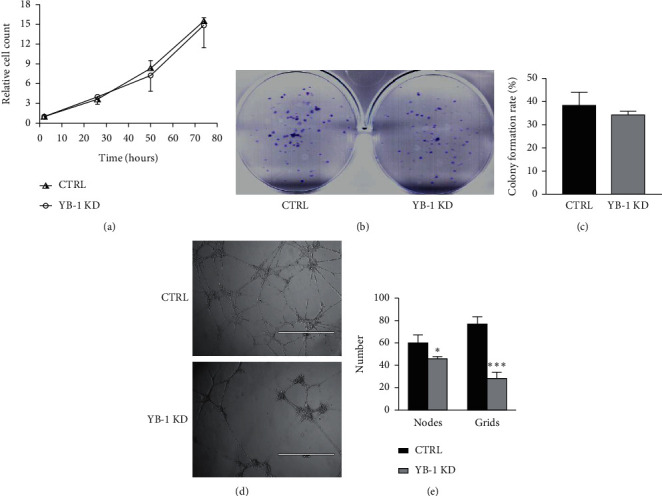
Evaluating the ability of CTRL-EJ and YB-1 KD-EJ cell lines in colony formation, proliferation, and inducing angiogenesis. (a) Evaluation of colony formation ability of CTRL-EJ and YB-1 KD-EJ cells by CCK‐8 assay. (b, c) Colony formation assay of CTRL-EJ and YB-1 KD-EJ cells. (d, e) Determination of YB-1 effect on the ability of EJ cells to induce angiogenesis by tube network formation assay. All the grids and nodes of the tube network in the containers were counted for statistics. CTRL, control; YB-1 KD, YB-1 knockdown; CCK8, Cell Counting Kit‐8; nodes, nodes of tube network; grids, grids of tube network;  ^*∗*^*P* < 0.05,  ^*∗∗*^*P* < 0.01, and  ^*∗∗∗*^*P* < 0.001.

To explore the effect of YB-1 on BC, this work evaluated the ability of CTRL-EJ and YB-1 KD-EJ cell lines to form colonies, proliferate, and induce angiogenesis. CCK8 assay results showed a trend that the proliferation ability of YB-1 KD-EJ cells was lower than that of CTRL-EJ cells, but there was no statistical significance (Figure 3(a)). Colony formation assay results showed that CTRL-EJ and YB-1 KD-EJ cells had similar colony formation ability with mean of colony formation rates of CTRL-EJ and YB-1 KD-EJ cells being 38.5 and 34.3, respectively (Figures 3(b) and 3(c)). To investigate the role of YB-1 in the ability of EJ cells to induce angiogenesis, tube network formation assay was performed using EA.hy926 cells in the special containers described in the Methods section. All the grids and nodes of the tube network in the containers were counted. Besides, the tube network in the CTRL-EJ group was significantly denser than that in the YB-1 KD-EJ group (Figure 3(d)). The statistics showed that grids and nodes of the tube network in the YB-1 KD-EJ group were 25% and 50% less than that in the CTRL-EJ group, respectively (Figure 3(e)). Results showed that the ability of EJ cells to induce angiogenesis, rather than that of colony formation and proliferation, was weakened after YB-1 was knocked down.

### 3.4. The Expression of VEGFA Increased When miR-29b-3p Was Inhibited in YB-1 KD- EJ Cells

**Figure 4:**
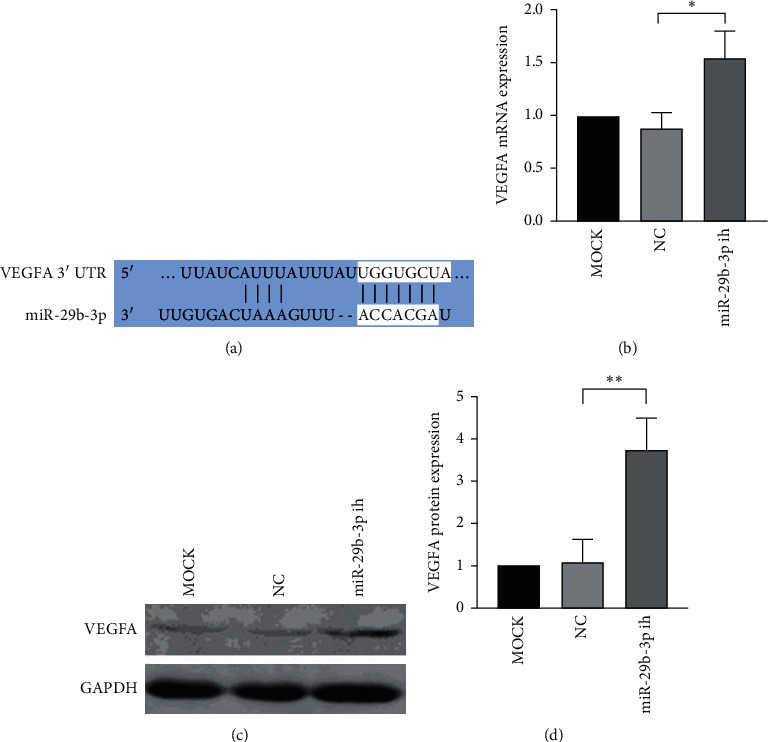
VEGFA was confirmed as one of the target genes of YB-1 by experiments and bioinformatics analysis. (a) miR-29b-3p may bind the sequence (UGGUGCU) in 3′ UTR of VEGFA to function according to TargetScanHuman 7.1. (b–d) miR-29b-3p was inhibited by miRNAs inhibitor in YB-1 KD-EJ cells. Then, the relative expression level of VEGFA was measured by RT-qPCR and Western Blot. YB-1 KD, YB-1 knockdown; 3′ UTR, 3′‐untranslated region; NC, negative control; miR, microRNA; ih, inhibitor;  ^*∗*^*P* < 0.05 and  ^*∗∗*^*P* < 0.01.

As mentioned above, the expression of VEGFA was downregulated, while that of miR-29b-3p was upregulated after YB-1 was knocked down in two BC cell lines. What is more, VEGFA was predicted as one of the target genes of miR-29b-3p in TargetScanHuman 7.1(http://www.targetscan.org). Moreover, miR-29b-3p might bind the sequence (UGGUGCU) in 3′ UTR of VEGFA to function (Figure 4(a)). Thus, miR-29b-3p was inhibited to check if the expression of VEGFA could be reversed in YB-1 KD-EJ cells. As a result, the expression of VEGFA was upregulated after miR-29b-3p was inhibited (Figures 4(b)–4(d)).

### 3.5. After YB-1 Knockdown, the BC Model Growth Slowed Down and the Microvessel Density of Tumors Decreased In Vivo

**Figure 5:**
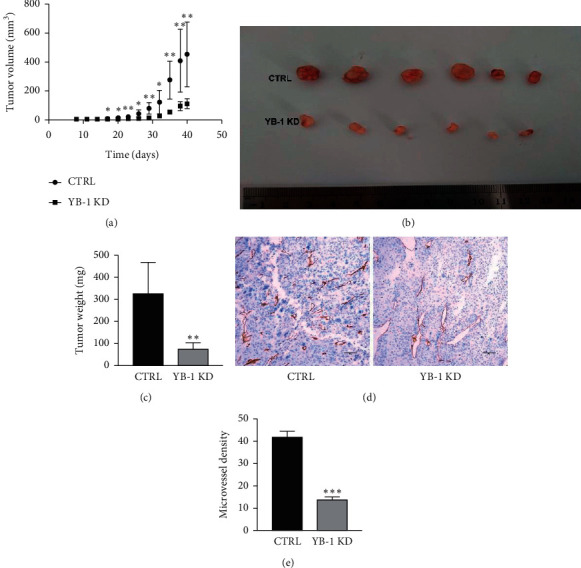
Effect of YB-1 on tumor growth and angiogenesis in vivo. (a) Tumor growth curve. From the eighth day after the inoculation, the tumor volume was measured by an external caliper every three days. (b, c) Tumors' picture and weight when they were harvested. (d, e) The microvessel density in these BC models was evaluated by immunohistochemistry. The brown circles in the picture represent the microvessels. The circles, in a field of view (100×), were counted for statistics. CTRL, control; YB-1 KD, YB-1 knockdown;  ^*∗*^*P* < 0.05,  ^*∗∗*^*P* < 0.01, and  ^*∗∗∗*^*P* < 0.001.

A total of 12 male BALB/c nude mice were subcutaneously injected with CTRL-EJ cells or YB-1 KD-EJ cells randomly. After eight days, all the mice had visible tumors at the injection site. However, the tumors of YB-1 KD-EJ cells grew slower than those of CTRL-EJ cells. The average tumor weights of CTRL group and YB-1 KD-EJ group were 327.3 mg and 75.5 mg, respectively (Figures 5(a)–5(c)). Furthermore, the tumor microvessels in the CTRL-EJ group were significantly denser than those in the YB-1 KD-EJ group. The mean microvessel density of CTRL group and YB-1 KD-EJ group was 42.1 and 14 per field, respectively (Figures 5(d) and 5(e)). Results indicated that YB-1 promotes the growth and angiogenesis of BC in vivo.

## 4. Discussion

As an oncoprotein, YB-1 has been reported to relocate to the nucleus to promote tumor development and extensively to regulate the expression of VEGFA in various mechanisms at transcription levels [15,16]. However, YB-1 is arguably a cytoplasmic protein, primarily playing a role in cytoplasm. Speculatively, there may be a mechanism of YB-1 regulating VEGFA expression in the cytoplasm. Amal et al. confirmed that YB-1 enhances the translation of hypoxia-inducible factor 1 alpha (HIF1A) by directly binding mRNA coding HIF1A, which strongly promotes the transcription of VEGFA [17]. Except this, there are few reports of YB-1 regulating VEGFA expression in the cytoplasm. In this work, we discovered that YB-1 promotes the expression of VEGFA by blocking the biogenesis of miR-29b-3p in BC [8]. This might explain the extensive regulation of VEGFA by YB-1. Arguably, for the first time, we reported the effect of YB-1 on VEGFA from the perspective of regulating miRNA.

Angiogenesis plays an important role in the development of BC. For instance, the theoretical basis of narrow-band imaging cystoscopy for BC is that the microvessel density in BC tissue is higher than that in normal field [18]. Moreover, VEGFA, an important factor promoting angiogenesis, is one of the tumor markers for screening BC and predicting tumor lymphatic metastasis [13, 19]. At present, antiangiogenic drugs are majorly monoclonal antibodies to VEGF or VEGFR and are still in the clinical trial stages for BC. The trials revealed that the curative effect is unsatisfactory. Current research combined these antiangiogenic drugs with chemotherapy drugs to generate better outcomes [20]. Several lines of evidence have shown that YB-1 is implicated in the angiogenesis of many tumors, yet it has been rarely reported in BC [15]. We performed IHC in 56 BC specimens and discovered that specimens with high expression of YB-1 expressed VEGFA more than those with low expression of YB-1. This suggested that YB-1 might be implicated in the angiogenesis of BC. Moreover, YB-1 is involved in the drug resistance mechanism of BC [4, 21]. YB-1 may be involved in both drug resistance and angiogenesis in BC, and studies on YB-1 might have significant potential value in the treatment of BC.

Sufficient evidence suggests that YB-1 binds to noncoding RNAs in cells, but there is limited evidence that YB-1 affects the expression of related mature miRNAs. Blenkiron et al. reported that YB-1 binds to the precursors of let-7 and miR-320, but knocking down YB-1 did not change mature miRNAs expression levels in breast cancer cell lines [22]. In contrast, Shuai-Lai et al. found that YB-1 could not only bind miRNAs precursors, but also change the expression of miR-29b-3p, let-7-3p, and other miRNAs in glioblastoma cell lines. They further confirmed that YB-1 extensively binds to the terminal loop region of pri/pre-miR-29b-2 and regulates the biogenesis of miR-29b-2, which can be processed into miR-29b-3p [8]. The different conclusions may be attributed to the different cell lines and platforms used in the experiments. For example, different cell lines might have different posttranslational modifications of YB-1, such as the levels of phosphorylation. Then, the YB-1 with different posttranslational modifications functions differently, resulting in different capabilities in binding to miRNAs precursors [23–25]. Nevertheless, changes in miRNAs omics after YB-1 knockout or knockdown in BC cell lines remain understudied. Our study demonstrated that knocking down YB-1 resulted in a roughly twofold upregulation in the expression of miR-29b-3p in EJ and UMUC3 cell lines. This suggested that YB-1 might bind to pri/pre-miR-29b-2 and block the biogenesis of miR-29b-2 in BC. Undeniably, whether YB-1 is universal for involving in the formation of miRNAs in BC warrants further study on RNA immunoprecipitation and miRNA chip.

miR-29b is a number of miR-29 family, majorly reported as a tumor suppressor in many studies, and has been shown to mediate multiple oncogenic processes, including metabolism, proliferation, metastasis, and angiogenesis [26, 27]. This study found that YB-1 downregulates the expression of miR-29b-3p. Moreover, we noted an increase in VEGFA when miR-29b-3p was inhibited. Besides VEGFA, the inhibition of miR-29b could upregulate the expression of other oncoproteins, such as DNMT3b, LOXL2, and MMP2 in some cancers [28–30]. Therefore, the reduction of miR-29b-3p might regulate the development of BC and is an important target in the treatment of BC. Various miRNAs replacement therapies exhibit significant potential in treating BC. However, a few obstacles remain to be surmounted [31, 32]. This study indicated that the expression of miR-29b-3p in BC cells is upregulated by inhibiting the interaction between pri/pre-miR-29b-2 and YB-1. This provides a novel perspective on miRNAs replacement therapy [33]. For instance, by referring to YB-1, we may make some drugs to increase the contents of tumor-suppressing miRNAs in tumor cells.

Reports indicate that YB-1 promotes or inhibits the proliferation of tumor cells; however, the mechanism of this dual effect remains unclear. Its effect on proliferation may depend on the cell type [34]. After knocking down YB-1 in TCCsup and KK47 BC cell lines, Lyabin et al. identified a significant decrease in the cell proliferation capability [35]. Here, after knocking down YB-1 in EJ cells, we did not find a significant change in proliferation ability. Additionally, based on the colony formation ability of EJ cells, no significant decrease was noted in the clonal formation rate after the YB-1 knockdown. In vitro and in vivo experiments demonstrated that the ability of EJ cells to induce angiogenesis diminished significantly after YB-1 knockdown. Moreover, the growth of the tumor model slowed down after the YB-1 knockdown. Based on the above, we suggest that the ability of the tumor to induce angiogenesis was impaired after the knockdown of YB-1, causing nutritional deficiency and further inhibiting tumor growth.

## 5. Conclusions

In this study, we confirmed a hypothesis that YB-1 promotes the expression of VEGFA by downregulating miR-29b-3p in BC cell lines. Moreover, YB-1 promotes angiogenesis of BC in vitro and in vivo. Eventually, we deduced that YB-1 regulates angiogenesis in BC via the miR-29b-3p-VEGFA pathway. This may help us understand the mechanism of YB-1 promoting the development of BC and provide evidence for YB-1 as a therapeutic target of BC. miRNAs replacement therapies harbor a significant potential for BC therapy. Inspired by the mechanism and effect of YB-1 to regulate miR-29b-3p, some drugs can be administered to increase the contents of tumor-suppressing miRNAs in tumor cells by referring to YB-1. Therefore, further investigations on the details of YB-1 binding to pri/pre-miRNA are essential.

## Data Availability

The data used to support the findings of this study are included within the article.

## Supplementary Materials

Supplementary Materials Table 1: sequences of primers.
