# Supplementary material for: Y-Box Binding Protein 1 Regulates Angiogenesis in Bladder Cancer via miR-29b-3p-VEGFA Pathway
Source: J Oncol. 2021 Jul 1;2021:9913015. doi: 10.1155/2021/9913015 (PMC8270724; doi:10.1155/2021/9913015)
Supplement: Supplementary Materials — Table 1: sequences of primers. [file 9913015.f1.docx]

| YB-1 | forward | GGAACGGATATGGTTTCATCAACA |
| --- | --- | --- |
|  | reverse | CCTTCAACAACATCAAACTCCACAG |
| VEGFA | forward | TGCCTTGCTGCTCTACCTCC |
|  | reverse | GCTGCGCTGATAGACATCCAT |
| GAPDH | forward | GCCACCCAGAAGACTGTGGAT |
|  | reverse | GGGATGACCTTGCCCACAG |
| hsa-miR-29b-3p | miRNA-specific 5′ primer | CCGTAGCACCATTTGAAATCAGTGTT |
| U6 | forward | Provided by Takara reagent |
|  | reverse |  |
| mRQ 3′ Primer | miRNA 3′ primer | Provided by Takara reagent |

Table I: Sequences of primers
